# Obstructive Sleep Apnea (OSA) and Patent Foramen Ovale (PFO), the “Perfect Storm” for a Cryptogenic Stroke? Case Series of Four Patients

**DOI:** 10.3389/fstro.2022.916154

**Published:** 2022-09-21

**Authors:** Alexandra Schwarz, Albert Lee, Nancy E. Gadallah, Jessalyn Wong, Divya Gupta

**Affiliations:** ^1^JFK Neuroscience Sleep Center, JFK Medical Center, Edison, NJ, United States; ^2^Department of Neurology, Hackensack Meridian School of Medicine, Edison, NJ, United States; ^3^Hackensack Meridian School of Medicine, Nutley, NJ, United States

**Keywords:** patent foramen ovale, stroke, obstructive sleep apnea, sleep apnea, cryptogenic stroke

## Abstract

We were intrigued by the finding of severe obstructive sleep apnea (OSA) in patients referred to our Sleep Clinic with a history of stroke at a relatively young age, in the setting of a patent foramen ovale (PFO). This prompted us to do a literature search to see the association of OSA with stroke in such a patient population. The correlation of PFOs with cryptogenic strokes is well established, as is that of OSA with stroke. However, not many studies have looked at the correlation between OSA and PFO in patients with cryptogenic strokes.

## Methods

We conducted a retrospective chart review of patients seen in our Sleep Clinic with a history of stroke and PFO and who were found to have OSA.

## Introduction

We were intrigued by the finding of severe obstructive sleep apnea (OSA) in patients referred to our Sleep Clinic with a history of stroke at a relatively young age, in the setting of a patent foramen ovale (PFO). This prompted us to do a literature search to see the association of OSA with stroke in such a patient population. The correlation of PFOs with cryptogenic strokes is well-established, as is that of OSA with stroke. However, not many studies have looked at the correlation between OSA and PFO in patients with cryptogenic strokes.

During fetal development, a PFO is essential as fetuses rely on the right to left shunting (RTLS) for oxygenation as they receive oxygen *via* the venous system. Soon after birth, the foramen ovale closes for the majority of the population as the RTLS is no longer needed. PFO persists in about 15–34% of the general population and is considered a normal variant (Guchlerner et al., 2012; Kar, 2014). Most remain asymptomatic, but in some people, PFO may be responsible for cryptogenic stroke. The risk of stroke in the general population is 24.9% (Virani et al., 2020); PFO is found in 20–54% of patients with strokes, and ~40% of ischemic strokes in adults under 55 are cryptogenic (Lamy et al., 2002).

Obstructive sleep apnea is found in about 9% of women and 24% of men (Barone and Krieger, 2013; Kar, 2014). OSA increases the risk of stroke by 2-fold in those with severe OSA. One of the proposed mechanisms for the increased risk is through dynamic changes in sleep creating RTLS. Studies have shown a >2-fold increase in RTLS in patients with OSA and PFO compared to patients with PFO alone (Barone and Krieger, 2013). OSA patients with PFO tend to experience significant nocturnal desaturation out of proportion to the respiratory event thought to be due to the mixing of deoxygenated blood from the venous to the arterial system (Shanoudy et al., 1998; Barone and Krieger, 2013). Hence, there is an increased risk of cardio-embolic stroke in such patients.

Patients with OSA also have a significantly higher risk of stroke and venous thrombus embolism (VTE) (Guchlerner et al., 2012; Alonso-Fernández et al., 2020). The risk worsens with the severity of the OSA. Patients with OSA were strongly associated with cardioembolism compared to other stroke etiologies even when controlled for atrial fibrillation (Lipford et al., 2015).

Patent foramen ovales are more commonly seen in patients with OSA compared with patients without OSA (69 vs. 17%) (Shanoudy et al., 1998; Beelke et al., 2003). Patients with OSA also tend to have a greater prevalence of RTLS when PFO was present (Beelke et al., 2003; Mojadidi et al., 2015).

There are several proposed mechanisms for the increase in RTLS seen in OSA (**Figure 2**). An increase in negative intrathoracic pressure (as seen during apneic episodes when a patient attempts to breathe against a closed glottis) causes a steady increase in right atrial pressure (RA) at which point the RA exceeds left atrial (LA) pressure (Beelke et al., 2002; Konecny et al., 2012). Another proposed mechanism is that apnea-induced hypoxemia and hypercapnia increase pulmonary arteriolar resistance and vasoconstriction, causing the transient elevation in pulmonary arterial pressure and subsequently increased right-sided pressure. The degree of desaturation during obstructive apneas tends to increase with those with RTLS compared to those without as seen with greater O_2_ desaturation during Valsalva (Beelke et al., 2002; Virani et al., 2020). In a case series, patients with cryptogenic strokes and OSA were found to have severe hypoxemia (Man et al., 2019). It was also found that patients with OSA and PFO had significantly increased estimated pulmonary artery systolic pressure (32 vs. 22 mmHg) during Valsalva (Shanoudy et al., 1998; Beelke et al., 2002, 2003). Therefore, in the presence of a PFO, the increase in RA pressure can create a right to left shunt possibly sending emboli from the right atrium to the left atrium. Multiple studies have shown that microemboli detected during apneic episodes correlated with the number of microemboli seen during the Valsalva maneuver (Beelke et al., 2002, 2003). A decrease in the recurrence of an embolic stroke may be seen if patients are diagnosed and treated for their sleep-disordered breathing as seen in other studies (Pinet, 2005).

Obstructive sleep apnea may also result in atrial cardiopathy, increasing the likelihood of stroke. Studies have suggested that a strong association exists between OSA and atrial fibrillation (AF), with the Sleep Heart Health Study showing a 4-fold increase in the prevalence of AF in patients with OSA (Mehra et al., 2006). OSA is associated with autonomic dysfunction, which could promote atrial fibrillation and atrial cardiopathy. An increase in vagal tone during apnea could shorten atrial refractory periods, while the increase in sympathetic tone at the termination of apnea could lead to enhance automaticity and trigger AF (Siontis and Oral, 2017). In addition, OSA could drive structural atrial alterations as well as an increased thrombotic state *via* mechanisms including dysregulation of the autonomic nervous system and cytokines (Acampa et al., 2018). Atrial cardiopathy could then increase the risk of AF and thromboembolism, resulting in a stroke. Though the heart is the origin of the stroke in this mechanism, the stroke may be mistakenly called cryptogenic if a patient has atrial cardiopathy without AF (Kamel et al., 2016).

We present a case series of four patients with PFO who presented with strokes and were found to have comorbid OSA.

## Case Description

### Patient 1

A 39-year-old right-handed woman with a history of migraines and obesity experienced a sudden onset of left-sided weakness. The patient had no prior history of stroke. Family history negative for stroke or myocardial infarction. Social history is pertinent for a 13-year history of smoking one pack per day. Admission ECG was normal with blood pressure elevated to 146/75. A transcranial Doppler (TCD) of the left middle cerebral artery with bubble study showed evidence of moderate to severe RTLS. CT angiography (CTA) of the head and neck was unremarkable for any large vessel occlusion or vascular stenosis. A transesophageal echocardiogram (TEE) revealed an atrial septum aneurysmal, and no thrombus was visualized. The heart monitoring device did not reveal cardiac arrhythmia. Hypercoagulable workup, homocysteine, protein c and s, antithrombin III, and activated protein c resistance, were negative. She was found to have a right parietal cortical infarct and was started on anticoagulation therapy with apixaban 5 mg twice a day until her PFO closure followed by 6 months of dual antiplatelet therapy. Imaging did not show any small vessel ischemic disease. Exactly 2 months post-stroke, the patient was referred for a polysomnogram due to snoring and witnessed apneas which showed moderate OSA overall with AHI (apnea-hypopnea index) of 17.6/h, which was severe in REM sleep with AHI of 50.1/h. O_2_ saturation nadir of 84%, and O_2_ saturation <90% for 1% of the total sleep time. Three months post-stroke, the patient underwent PFO closure. Exactly 4 months post-stroke, the patient was placed on CPAP pressure of 12 cm H_2_O per the recommendation of the CPAP titration study. At the following clinic visit 1 month after starting CPAP therapy, the patient achieved adequate adherence and efficacy with CPAP with usage 6 h a night for 100% of the last 30 nights and residual AHI of 0.6/h. Adequate adherence is assessed by the use of CPAP for >70% of the nights for at least 4 h and with a residual AHI of <5/h (Centers for Medicare Medicaid Services, 2021). The patient tolerated CPAP well. As of 2 years later, the patient has not had a recurrence of the stroke (Tables 1, 2).

**Table 1:** Patient demographics.

**Patient**	**Age when** **stroke** **occurred**	**Sex**	**Hypertension**	**Diabetes** **mellitus**	**Dyslipidemia**	**Smoking** **history**	**Body mass** **index** **(kg/m^2^)**	**Coronary** **artery** **disease**	**Migraine**	**Hypercoagulable** **work up**
1	38	Woman	No	No	Yes	Smoker	49.4	No	Yes	Negative
2	46	Woman	Yes	No	Yes	Smoker	47.2	No	No	Negative
3	59	Man	No	No	No	Smoker	25.4	No	No	Not available
4	68	Man	Yes	Yes	No	Former (quit smoking 30 years ago)	33.1	No	No	Not available

**Table 2:** Sleep test results of patients in the case series, in regards to sleep apnea.

**Patient**	**Echocardiogram**	**Pulmonary** **artery** **pressure**	**Right atrial** **pressure**	**Left atrial** **pressure**	**Magnetic resonance imaging** **of the brain**	**Transcranial** **doppler with** **bubble**	**Transesophageal** **echocardiogram** **(TEE)**	**Sleep test results**
1	The left ventricular ejection fraction (estimate) 50–55%. The right ventricular systolic function is normal.	28 mmHG (normal)	3 with >50% variation with respiration (normal)	11 mmHg (normal)	1 cm focus of recent ischemic infarct of the right posterior frontal lobe.	Moderate to severe RTLS spontaneously and with valsalva.	PFO noted with moderate R–>L shunt, even without valsalva maneuver.	Polysomnography: moderate obstructive sleep apnea (OSA) with apnea+hypopnea index (AHI) of 17.6/h in general, and severe in supine REM sleep with AHI 50.1/h. O_2_ saturation nadir of 84%, and O_2_ saturation <90% for 1.0% of the total sleep time.
2	The left ventricular ejection fraction (estimate) 45–50%.	Not reported.	Not well-visualized	6 mmHg (normal)	Small patchy area of restricted diffusion within the lateral right frontoparietal region compatible with an acute to subacute infarct.	Positive (Grade IV 101–300)	A PFO is present. Valsalva maneuver was not reported	Home Sleep Test (WATCHPAT device): severe obstructive sleep apnea, in general, with p Apnea+hypopnea Index (pAHI) of 36.33 per hour, and worse in REM sleep at 55/h. O_2_ saturation nadir of 85%, O_2_ saturation were <90% for 0.8% of total sleep time.
3	The left ventricular ejection fraction (estimate) 50–55%.	30.9 mmHg (normal)	10 mmHg (intermediate)	8.4 mmHg (normal)	Acute left middle cerebral artery infarct.	Not available	Probable tiny PFO with minimal right to left shunting (report was not available) Results was obtained from old notes	Polysomnography: mild OSA overall with AHI of 6.1, but severe OSA in REM sleep with AHI 29.6/h. O_2_ nadir saturation was 88%, O_2_ sats were <90% for 1% of the total sleep time
4	The left ventricular ejection fraction (estimate) 55–60%.	32.2 mmHg (normal)	10 mmHg (intermediate)	8.3 mmHg (normal)	Patchy acute to subacute left superior cerebellar infarct	Not available	A PFO is present. Vasalva maneuver was not reported.	Polysomnography: mild OSA with AHI 14.0/h in general, though severe in supine sleep with AHI of 80.5/h. O_2_ saturation nadir of 90%.

### Patient 2

A 46-year-old woman with a history of obesity, hypertension, hyperlipidemia, and hypothyroidism on oral contraceptive pills presented with left hemiparesis and hemiparesthesia. Her social history was pertinent for her current smoking ½ pack per day. Her family history is pertinent to stroke in her father at an early age. On admission, her blood pressure was elevated to 169/58 and her ECG showed normal sinus rhythm with possible anterior infarct, age undetermined. MRI of the brain showed small patchy acute to subacute infarcts in the lateral right frontoparietal region. CTA of the head and neck did not show any large vessel occlusion. TCD with bubble study was positive and hence indicated an RTLS. A TEE showed a PFO and mild atherosclerotic plaque in the descending aorta with no thrombus or aneurysmal visualized. Venous Doppler of lower extremities was negative for deep vein thrombosis. The patient was started on anticoagulation therapy with apixaban. Hypercoagulable workup including Beta2 GP, Protein C and S, Factor V activity, LAC, Anticardiolipin, ANA, and Antithrombin III was unrevealing. The Loop recorder showed an ectopic beat but no atrial fibrillation. Her PFO was closed 4 months later. Six months post-stroke, a home sleep study showed severe OSA with AHI of 36.3, worse in REM sleep with AHI of 55/h. O_2_ saturation nadir of 85%, O_2_ saturation were <90% for 0.8% of total sleep time. Due to the COVID-19 pandemic, the patient opted for auto-CPAP (8–20 cm H_2_O) which she received 1 week after her sleep study. At the following clinic visit 1 month after starting CPAP therapy, the patient achieved adequate adherence and efficacy with CPAP with the usage of 5.25 h a night for 100% of the last 30 nights and residual AHI (apnea-hypopnea index) of 1.8/h. She initially reported difficulty with adherence due to aggravation of temporomandibular joint dysfunction from her mask, however, symptoms resolved with a change in the mask. As of 2 years later, the patient has not had a recurrence of a stroke (Tables 1, 2).

### Patient 3

A 59-year-old man with hyperlipidemia experienced sudden onset of right-sided weakness following a brief period of bending over in his garden 2 days after a 5 h flight. Family history is significant for multiple cerebrovascular accidents in his father and hemorrhagic stroke in his sister. Social history is significant for being a current everyday smoker. He smoked one pack every other day since the age of 15 years. CT of the head showed a left frontoparietal stroke. MRI showed an acute left MCA infarct. The patient was admitted to an outside hospital for his stroke therefore admission vitals, EKG, and hypercoagulability workup are unknown. He was seen post-stroke for acute rehabilitation at our institution. MR angiography and carotid ultrasound were negative for large vessel occlusion and stenosis. A TEE showed probable small PFO with minimal RTLS and no evidence of thrombus. A TCD has been done 3 months post-stroke and showed no focal stenosis but did show holocephalic diminished systolic upstroke. The patient underwent a polysomnography 4 months post-stroke due to snoring and spontaneous nocturnal awakenings, which showed mild OSA overall with AHI of 6.1, but severe OSA in REM sleep with AHI of 29.6/h. O_2_ saturation nadir was 88%, and O_2_ sats were <90% for 1% of the total sleep time. Five months post-stroke, the patient was started on CPAP 12 cm H_2_O following recommendations from a CPAP titration study. IVC filter was also placed 5 months post-stroke. At the following clinic visit 6 months post-stroke, the patient self-reported adequate compliance with the usage of 8–9 h a night for 100% of the last 30 nights. The first compliance report available was 6 years post-stroke with usage for 8 h 100% of the night over the last 30 days with residual AHI of 0.4/h. The patient opted against PFO closure in favor of antiplatelet therapy. To date, the patient has not had a recurrence of the stroke (Tables 1, 2).

### Patient 4

**Figure 1:**
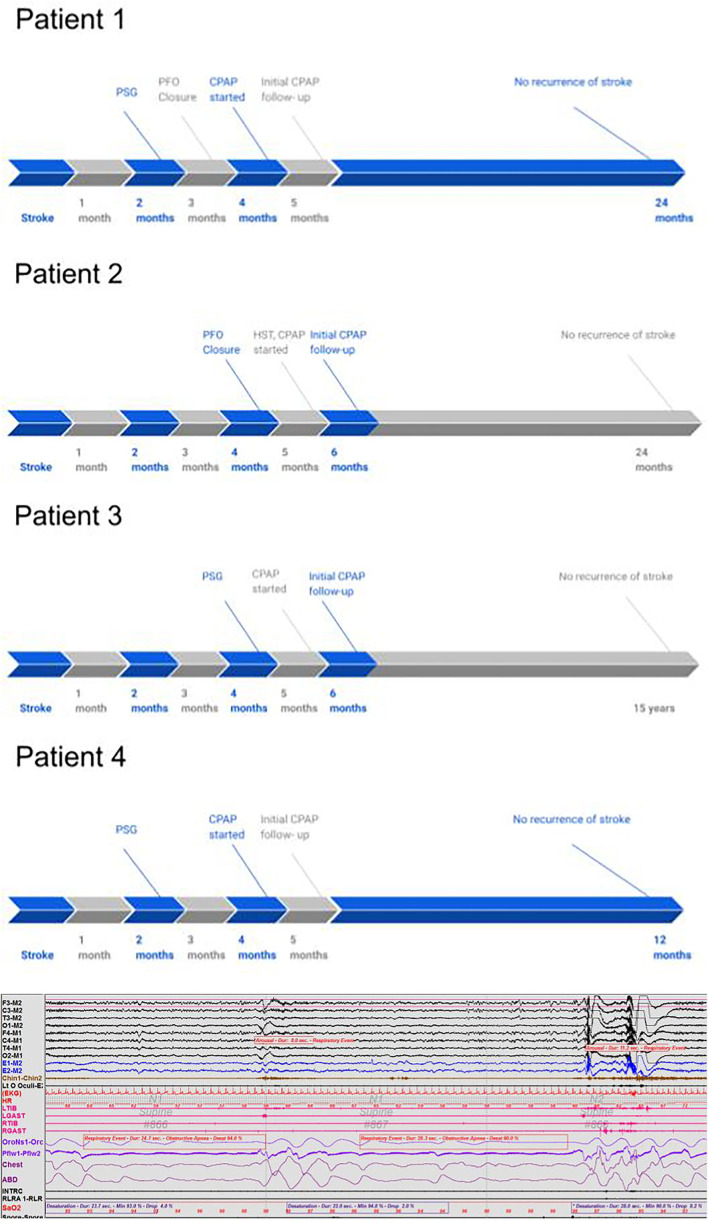
A 90-s polysomnography segment in a 68-year-old right-handed man with a history of hypertension, hypothyroidism, and pre-diabetes. The segment illustrates the impact of obstructive apnea events on cardiovascular and EEG parameters, such as causing O_2_ desaturation, heart rate elevation, and electroencephalogram arousal.

A 68-year-old man with HTN, hypothyroidism, pre-diabetes, and OSA (diagnosed in 2005 and non-adherent to CPAP as of 2018) on baby aspirin presented with imbalance, vertigo, and left-sided numbness. Social history is significant for the remote history of smoking. Admission blood pressure was controlled and EKG showed normal sinus rhythm. Brain MRI showed a patchy acute to subacute left superior cerebellar infarct with no evidence of small or large vessel disease. Head and neck Magnetic Resonance Angiography (MRA) showed an attenuated left vertebral artery with no evidence of stenosis. Hypercoagulable workup was unknown. A TEE showed a small PFO and mild athrosceloritc plaques in the descending aorta with no thrombus or aneurysmal visualized. The patient was started on antiplatelet therapy and did not undergo PFO closure. Exactly 2 months post-stroke, the patient underwent an additional sleep study which showed mild OSA with an AHI of 14/h, which was severe when supine with an AHI of 80.5/h. O2 saturation nadir of 90%. Exactly 4 months post-stroke, a CPAP titration study was suboptimal as the patient did not have supine REM sleep. Therefore, auto-CPAP 11–15 cm was recommended. He received his CPAP machine 5 months post-stroke. A Loop recorder was also placed given high suspicion for atrial fibrillation and high suspicion for cardioembolism, however, results were unknown. At the following clinic visit, 5 months post-stroke, the patient achieved adequate adherence and efficacy with CPAP with the usage of 6.5 h a night for 90% of the last 30 nights and residual AHI (apnea-hypopnea index) of 1.9/h. As 1 year later the patient had no stroke recurrence (Tables 1, 2 and Figure 1).

## Discussion

These patients with stroke at a relatively young age had PFO and had severe OSA in REM sleep with prominent O_2_ desaturation accompanied by heart rate elevation and electroencephalogram (EEG) arousals. Similar findings have been reported in a case series with three young patients with “wakeup” stroke with severe OSA (Man et al., 2019). Since the cases that we have described were seen over a span of several years, we have Spencer grading available in only the more recent cases as seen in patient 2. A higher Spencer grade is associated with a greater RTLS (Spencer et al., 2004). Although Spencer grading has only recently been reported in TCD studies at our center, it is notable that patient 2 who had a high Spencer grading also had severe OSA in general.

Several of the subjects were also noted to have RTLS, even without Valsalva, which could worsen with OSA as seen on TCD. The two of the five subjects had moderately elevated right atrial pressures on echocardiogram. Subjects were started on positive airway pressure (PAP) therapy with no known re-occurrence of the stroke to date. Obesity in general increases the risk of both OSA and stroke. In our case series, patient 2 had a lower BMI with mild OSA and a small PFO with minimal shunting (Tables 1, 2). That patient opted not to have PFO closure with no recurrence of transient ischemic attacks (TIAs) and was well-managed with CPAP therapy.

It is thought that untreated OSA increased RTLS *via* the PFO leading to stroke by paradoxical thromboembolism. This is supported by the findings in our case series.

**Figure 2:**
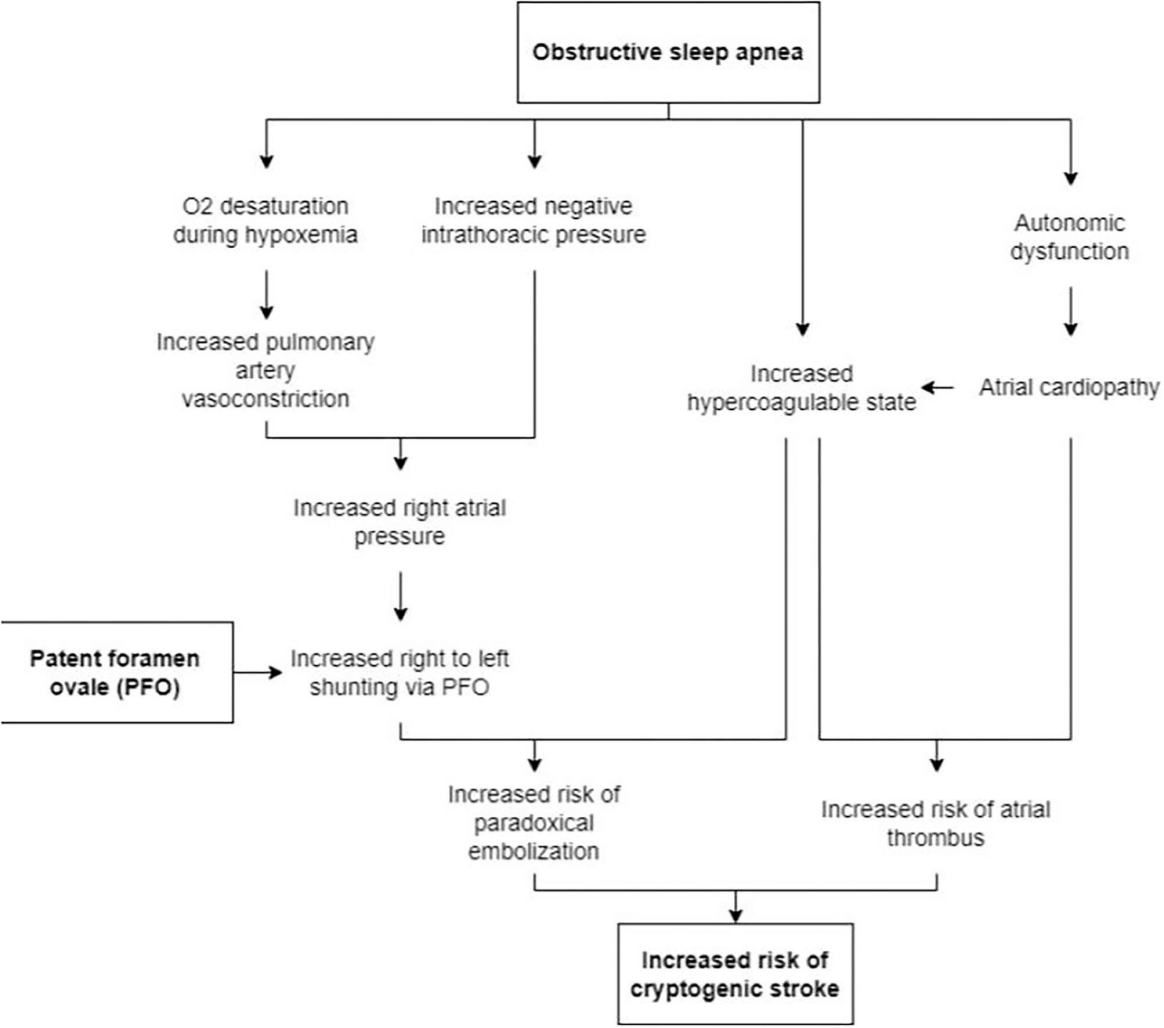
Proposed mechanisms for the increased risk of stroke in patients with PFO who also have OSA.

We have explained the trifecta of PFO, OSA, and stroke and the inter-related pathophysiological mechanisms in Figure 2, particularly the increase in right-to-left shunt in patients who have PFO.

## Conclusion

In conclusion, we suggest that PFO in OSA patients is a potential risk—a “perfect storm”- for cryptogenic stroke. Hence it is important to investigate and treat any comorbid OSA in those with PFO and stroke to reduce morbidity and mortality. American Academy of Neurology provided a level B recommendation to evaluate for OSA and monitor for atrial fibrillation for at least 1 month following cryptogenic stroke (Messé et al., 2020). Further studies including a large number of patients are needed to assess correlations among stroke, OSA, and PFO.

## Data availability statement

The raw data supporting the conclusions of this article will be made available by the authors, without undue reservation.

## Ethics statement

Written informed consent was obtained from the individual(s) for the publication of any potentially identifiable images or data included in this article.

## Author contributions

DG did the conception and design of the study. NG organized the database. AS and JW wrote the first draft of the manuscript. AS, JW, and AL contributed to manuscript revision, read, and approved the submitted version. AL contributed to the figure. All authors helped with the literature search. All authors contributed to the article and approved the submitted version.

## Conflict of Interest

The authors declare that the research was conducted in the absence of any commercial or financial relationships that could be construed as a potential conflict of interest.

## Publisher's Note

All claims expressed in this article are solely those of the authors and do not necessarily represent those of their affiliated organizations, or those of the publisher, the editors and the reviewers. Any product that may be evaluated in this article, or claim that may be made by its manufacturer, is not guaranteed or endorsed by the publisher.
